# Yap is essential for uterine decidualization through Rrm2/GSH/ROS pathway in response to Bmp2

**DOI:** 10.7150/ijbs.67756

**Published:** 2022-03-06

**Authors:** Hai-Fan Yu, Zhan-Qing Yang, Ming-Yue Xu, Ji-Cheng Huang, Zhan-Peng Yue, Bin Guo

**Affiliations:** 1 College of Veterinary Medicine, Jilin University, Changchun, P. R. China.; 2 College of Life Science, Northeast Forestry University, Harbin, P. R. China.

**Keywords:** Yap, Bmp2, Rrm2/GSH/ROS pathway, mitochondrial function, decidualization

## Abstract

Yap is required for ovarian follicle and early embryo development, but little information is available regarding its physiological significance in decidualization. Here we determine the effects of YAP on decidualization, mitochondrial function, cell apoptosis and DNA damage, and explore its interplay with Bmp2, Rrm2, GSH and ROS. The results exhibited that Yap was abundant in decidual cells and its inactivation impaired the proliferation and differentiation of stromal cells along with the deferral of G1/S phase transition, indicating Yap importance in decidualization. Bmp2 via Alk2 receptor promoted nuclear translocation of Yap where it might interact with Tead and then bind to the promoter of Rrm2 whose activation rescued the faultiness of differentiation program and attenuated oxidative DNA damage caused by Yap impediment. Meanwhile, Yap had an important part in the crosstalk between Bmp2 and Rrm2. Furthermore, inactivation of Yap resulted in an obvious accumulation of intracellular ROS followed by the abnormal GR activity and GSH content dependent on Rrm2. Replenishment of GSH counteracted the regulation of Yap inactivation on stromal differentiation and DNA damage with distinct reduction for intracellular ROS. Additionally, blockage of Yap caused the enhancement of stromal cell apoptosis and brought about mitochondrial dysfunction as indicated by the aberration for ATP level, mtDNA copy number and mitochondrial membrane potential concomitant with the opening of mitochondrial permeability transition pore, but these abnormalities were neutralized by GSH. Administration of mitochondrial antioxidant Mito-TEMPO rescued the fault of stromal differentiation conferred by Yap inactivation. Collectively, Yap was essential for uterine decidualization through Rrm2/GSH/ROS pathway in response to Bmp2.

## Introduction

Decidualization, where uterine stromal cells proliferate and differentiate into decidual cells, occurs in response to implanting embryo and is a key event during early pregnancy because its failure results in unexplained infertility, implantation failure and recurrent pregnancy loss 1,2. Increasing evidence supported an importance of bone morphogenetic protein 2 (Bmp2) in uterine decidualization 3,4. Mice lacking Bmp2 in uteri were infertile due to the incapability of stromal cells in undergoing the decidual response 4, but much remained elusive about how Bmp2 precisely governed this process.

Yes-associated protein (Yap), the key downstream effector of Hippo signaling pathway, has been implicated in the modulation of organ size, stem cell function, regeneration and tumor development by regulating the expression of target gene in interaction with DNA binding transcription partners, such as TEA domain (Tead) transcription factors 5-7. Several reports have pointed out that Yap was closely related to female reproduction. Disruption of Yap caused an arrest for embryonic development at day 8.5 along with defects of yolk sac vasculogenesis as well as differentiation of embryonic stem cells 8,9. Inactivation of Yap by Verteporfin or deletion of Yap in granulosa cells impeded ovarian follicle development, whereas constitutively active Yap transgenic mice exhibited opposite effects 10,11. Meanwhile, deficiency of Yap in oocytes presented a slow progression of embryo development from two-cell through four-cell stage 12. Although abundant Yap protein has been detected in the nucleus of human decidual cells 13, little information is available regarding its physiological significance in uterine decidualization.

This present study revealed that Yap was essential for uterine decidualization in response to Bmp2. Inactivation of Yap resulted in the impairment of stromal differentiation through inducing mitochondrial dysfunction and cellular apoptosis dependent on ribonucleotide reductase M2 (Rrm2)/reduced glutathione (GSH)/reactive oxygen species (ROS) pathway.

## Materials and Methods

### Animal treatment

Female mice (aged 8-10 weeks, weighed 26 ± 1 g) were housed with fertile or vasectomized males to induce pregnancy or pseudopregnancy by cocaging, respectively (day 1= vaginal plug). To artificial decidualization model, 20 µl of sesame oil was injected into one uterine horn on day 4 of pseudopregnancy, while another non-oil-infused horn served as a control. The uteri were collected at 48, 72 or 96 h. All animal experiments were approved by the Committee for the Ethics on Animal Care and Use of Jilin University (SY201905031).

### Isolation and treatment of uterine stromal cells

As previously described 14,15, uterine stromal cells from day 4 of pregnancy were isolated by enzymatic digestion followed by purity analysis with vimentin (a marker for stromal cells) and cytokeratin (a marker for epithelial cells) staining and then cultured with DMEM-F12 containing 10% heat-inactivated FBS (Clark, #25015). Meanwhile, stromal cells were treated with 10 nM of estradiol-17β (Sigma, #E1024, 10 nM) plus 1 μM of progesterone (Sigma, #850454, 1 μM) in DMEM-F12 containing 2% charcoal-treated FBS (Gibco, #12676-029) to *in vitro* decidualization which was verified as previously described 15,16. For further analysis, cells were treated with Yap inhibitor Verteporfin (100 nm, R&D systems, #5305) in the absence or presence of recombinant murine Bmp2 protein (rBmp2, 100 ng/ml, Peprotech, #120-02), glutathione reductase (GR) inhibitor 1, 3-bis (2-chloroethyl)-1-nitrosourea (BCNU, 20 μM, Sigma, #154-93-8), GSH (1 mM, Beyotime, #S0073) and mitochondria-targeted antioxidant Mito-TEMPO (MCE, 10 mΜ, #HY-112879) under the context of *in vitro* decidualization. Additionally, stromal cells were incubated with rBmp2 in the absence or presence of Rrm2 inhibitor Triapine (10 μM, Selleck, #S7470). Estradiol-17β, progesterone, Verteporfin, BCNU and Mito-TEMPO were dissolved in ethanol or DMSO, while rBmp2 and GSH were dissolved in PBS. Controls received the vehicle only.

### *In situ* hybridization

As previously described 15, *in situ* hybridization was executed in frozen sections. After label of Yap cRNA probe with digoxigenin (DIG), cryosections were fixed with 4% paraformaldehyde and then hybridized for 16 h followed by the incubation of sheep anti-DIG antibody conjugated to alkaline phosphatase (1:5000, Roche, #11093274910) and visualization with 5-bromo-4-chloro-3-indolyl phosphate (BCIP, 0.4 mM, Amresco, #0885) and nitro blue tetrazolium (NBT, 0.4 mM, Amresco, #0329).

### Immunofluorescence

Decidual cells from day 7 of pregnancy were isolated as previously described 15 and then seeded in glass coverslip. After fixation and blockage with 3% BSA, cells were incubated with Yap antibody (1:100, Cell Signaling Technology, #4912) or Rrm2 antibody (1:100, Thermo Fisher Scientific, #PA5-92365) followed by the replenishment of Goat anti-Rabbit Alex Fluor 488 conjugated antibody (1:1000, Invitrogen, #A32731) and counterstained with DAPI. Images were obtained by confocal or fluorescence microscopy. Same concentration of normal rabbit IgG was used to replace Yap or Rrm2 antibody as a negative control.

### Western blot analysis

Total and nuclear proteins from homogenized uteri or cells were lysed with ice-cold RIPA buffer and subjected to SDS-PAGE followed by the transfer of proteins to PVDF membrance. After blocking with 5% skim milk, membranes were probed with antibody against Yap (1:1000, Cell Signaling Technology, #4912), phospho-Yap S127 (1:1000, Cell Signaling Technology, #4911) Rrm2 (1:1000, Thermo Fisher Scientific, #PA5-92365), B cell leukemia/lymphoma 2 (Bcl2, 1:1000, Proteintech, #12789-1-AP), Bcl2-associated X protein (Bax, 1:1000, Proteintech, #50599-2-Ig), or Cleaved Caspase 3 (Casp3) (1:500, Cell Signaling Technology, #9664) and then incubated with HRP-conjugated secondary antibody (#SA00001-2, Proteintech, 1:5000). Signals were detected by ECL Chemiluminescent kit. Gapdh (1:5000, Proteintech, #10494-1-AP) and Histone H3 (1:5000, Proteintech, #17168-1-AP) were used as the loading controls.

### Real-time PCR

Total RNAs were extracted from uteri or cells with TRIPURE reagent and then applied to synthesize cDNA. Real-time PCR was executed to evaluate the expression levels of different gene as described previously 15. All primers were listed in Table S1.

### RNA interference

Yap siRNAs were synthesized by GenePharma and listed as follows: GGACCUCUUCCUGAUGGAUTT (siRNA 1), CCAACCAGCAGCAGCAAAUTT (siRNA 2), and GGUUGAAACAACAGGAAUUTT (siRNA 3), while activin-like kinase 2 (Alk2) siRNA was referred 17. After transfection with Yap or Alk2 siRNA, stromal cells were gathered in the existence or not of rBmp2 under the context of *in vitro* decidualization.

### Plasmid construction

Rrm2 overexpression plasmid was constructed with specific primer (Table S1) as previously described 15, and then guided into stromal cells followed by an addition of Yap inhibitor Verteporfin under *in vitro* decidualization. Meanwhile, Yap siRNA and Rrm2 overexpression plasmid were introduced into stromal cells in the presence of estradiol-17β plus progesterone.

### Cell proliferation

After treatment with Yap siRNA or its inhibitor Verteporfin, stromal cells were cultured for 24 h under *in vitro* decidualization. Finally, 20 µl of 3-(4,5-dimethylthiazol-2-yl)-5-(3-carboxymethoxyphenyl)-2-(4-sulfopheny)-2H-tetrazolium, inner salt (MTS, Promega, #G3580) reagent was added followed by the incubation for 2-4 h. Absorbance was measured by Multi-mode reader.

### Cell cycle analysis

After treatment with Yap siRNA or its inhibitor Verteporfin, stromal cells were cultured for 24 h in the existence of estradiol-17β and progesterone followed by the fixation with 70% ice-cold alcohol and staining with PI/RNase staining buffer (BD Biosciences, #550825). Finally, cells were analyzed by flow cytometry.

### Analysis of ALP activity as well as 8-OHdG and ATP contents

After treatment as mentioned above, stromal cells were lysed to determine the activity of alkaline phosphatase (ALP, Beyotime, #P0321S) and evaluate the contents of 8-hydroxydeoxyguanosine (8-OHdG, Cusabio, #CSB-E10527m) and ATP (Beyotime, #S0027) in accordance with the responding assay kit.

### Dual luciferase assay

Rrm2 promoter sequence (-1148 to -875) contained Tead binding site or its mutant site was synthesized and inserted into pGL6 vector. After transfection with pGL6-Rrm2 or pGL6-Rrm2-mutant plasmid, stromal cells were treated with Yap inhibitor Verteporfin under *in vitro* decidualization. Meanwhile, pGL6-Rrm2 or pGL6-Rrm2-mutant plasmid was co-transfected with Yap siRNA. To determine the transcriptional activity of Yap/Tead, 8xGTIIC-luciferase plasmid (Addgene, #34615) and Yap or Alk2 siRNA were transfected into stromal cells in the absence or presence of rBmp2. Concurrently, Yap inhibitor Verteporfin was supplemented in uterine stromal cells transfected with 8xGTIIC-luciferase plasmid. After different treatment, luciferase activity was evaluated by dual luciferase reporter gene assay kit (Beyotime, #RG088M). The pRL-SV40 plasmid (Beyotime, #D2768) was used for data normalization.

### Determination of ROS level

After treatment as described above, stromal cells were incubated with fluorescent probe DCFH-DA (Beyotime, #S0033, 20 μM) or dihydroethidium (DHE, Beyotime, #S0063, 10 μM) at 37 °C, washed three times and analyzed by Multi-Detection Microplate Reader or flow cytometry to assess the levels of intracellular ROS and superoxide anion (O_2_^-^).

### Measurement of GPX and GR activities as well as GSH content

After different treatment, proteins were harvested to determine the activities of glutathione peroxidase (GPX) and GR and assess the content of reduced glutathione (GSH) as well as GSH/oxidized glutathione (GSSG) ratio in accordance with the responding assay kit (Beyotime, #S0058, #S0055 or #S0053).

### Analysis of mitochondrial membrance potential (MMP)

After treatment with Yap siRNA or its inhibitor Verteporfin in the existence or not of GSH, stromal cells were incubated with the JC-1 staining solution (Beyotime, #C2006) followed by the analyzation of flow cytometry. The ratio of red and green fluorescent intensities indicated the change for mitochondrial membrane potential. To visualize MMP change, stromal cells were incubated with another indicator TMRM (100 nM, ThermoFisher Scientific, #I34361) and then stained with Hoechst 33342 followed by the obtainment of images in fluorescence microscope.

### Opening of mitochondrial permeability transition pore (mPTP)

After treatment with Yap siRNA or its inhibitor Verteporfin in the existence or not of GSH, stromal cells were incubated with Calcein AM fluorescent probe (Beyotime, #C2009S) concomitant with the supplementation of fluorescence quenching solution for 25 min at 37 °C. Finally, cells were analyzed by flow cytometry to determine the opening of mPTP.

### Determination of mtDNA copy number

After treatment with Yap siRNA or its inhibitor Verteporfin in the existence or not of GSH, total DNAs were extracted and then applied to determine mitochondrial DNA (mtDNA) copy number by analyzing the ratio of mtDNA/nuclear DNA (ncDNA) as previously described 18.

### Cell apoptosis

After treatment with Yap siRNA or its inhibitor Verteporfin in the existence or not of GSH, apoptosis rates of stromal cells were tested by flow cytometry in the light of Annexin V-FITC apoptosis detection kit instructions (Beyotime, #C1062S). Meanwhile, Casp3 activity was determined using the corresponding assay kit (Beyotime, #C1116).

### Statistical analysis

The statistical analyses were performed using SPSS19.0 software. Comparison between two groups was performed by Independent-Samples T Test, while multiple comparisons were tested with one-way ANOVA. All data were shown as means ± SEM. P < 0.05 was regarded as statistically significant.

## Results

### Yap expression during decidualization

**Figure 1:**
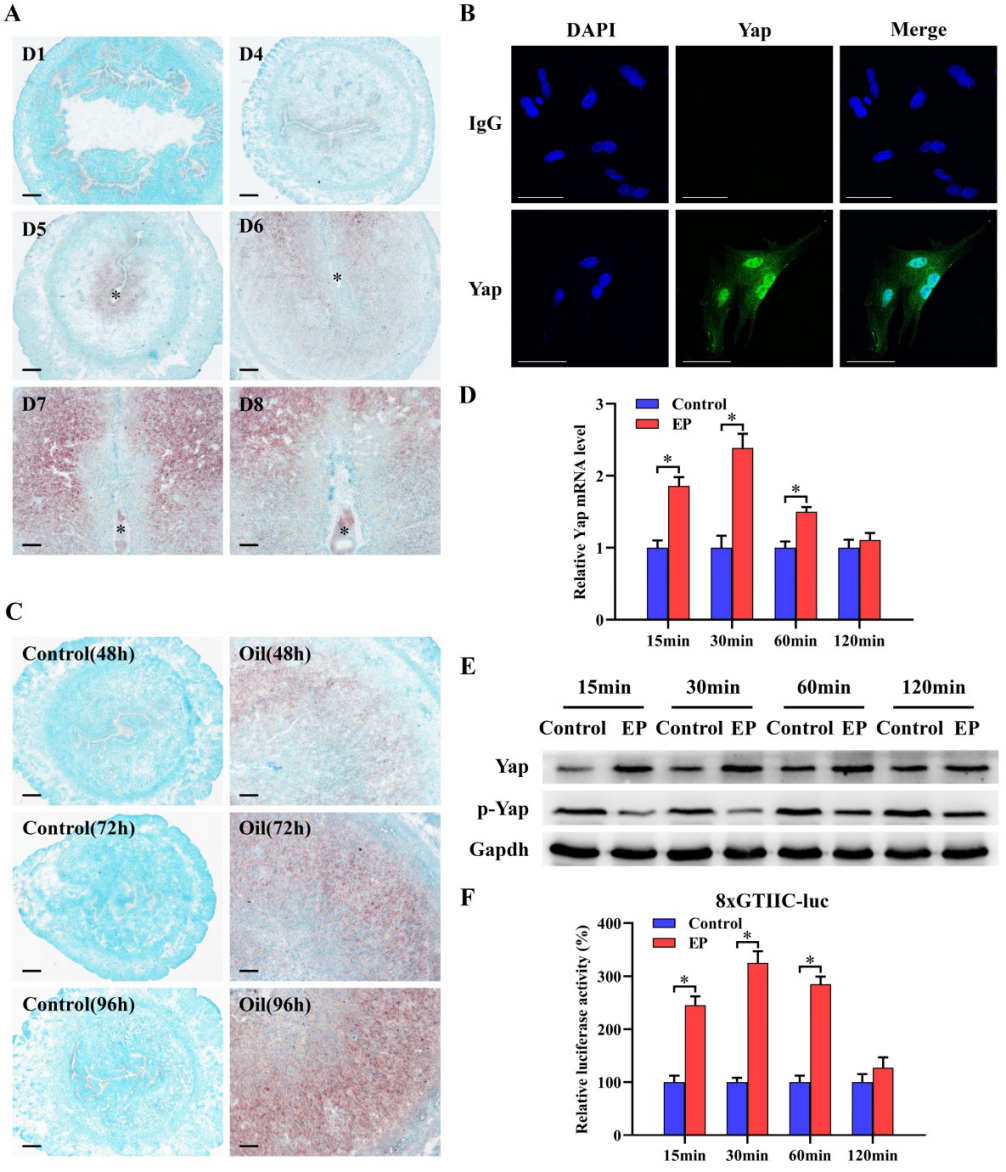
** Yap expression during decidualization. (A)** *In situ* hybridization analysis of *Yap* expression in pregnant mice uteri on days 1 and 4-8 (3 mice per group). Scale bar, 60 µm. Asterisks indicate embryo. **(B)** Immunofluorescence staining of Yap in uterine decidual cells from day 7 of pregnancy (3 mice per group). Scale bar, 60 µm. **(C)** *Yap* expression under artificial decidualization (3 mice per group). Control, uninjected uterine horn served as a control; Oil, oil-induced decidualization. **(D and E)** Yap mRNA and protein expression under *in vitro* decidualization (N = 3 per group). EP, estrogen plus progesterone. **(F)** Yap-Tead transcription activity under *in vitro* decidualization (N = 5 per group). Data are shown mean ± SEM. Asterisks denote significance (P < 0.05).

To explore the relevance between Yap and decidualization, we analyzed its expression in decidualized uteri and stromal cells. The results presented that faint signal for *Yap* mRNA was noted in the uteri on days 1-4 of pregnancy (Figure 1A). Followed by the initiation of embryo implantation, *Yap* mRNA was apparently noted in subluminal stroma at implantation sites from day 5 and became more visible in decidua from day 6 to 8 of pregnancy (Figure 1A). Real-time PCR and western blot analyses further confirmed abundant mRNA and protein levels for Yap in decidua, but not its phosphorylated level (Figure S1A and B). By immunofluorescence analysis, nuclear Yap protein was visualized in decidual cells from day 7 of pregnancy (Figure 1B). Compared to non-decidualized stromal cells, mRNA and total protein levels of Yap were elevated, whereas its phosphorylated level was significantly reduced (Figure S1C and D). Meanwhile, *Yap* mRNA signal was obviously localized in decidualizing stromal cells after sesame oil was injected into uterine lumen, but undetectable in control uteri (Figure 1C). Consistently, further quantitative analysis of Yap also revealed the enhancement of mRNA and total protein levels in oil-treated uteri, while its phosphorylated level exhibited the opposite effects (Figure S1E and F). Under *in vitro* decidualization, *Yap* mRNA levels showed an increase at 15 and 30 min, with a peak at 30 min followed by a decline reaching a level similar to that of control at 120 min (Figure 1D). By western blot analysis, Yap total protein was remarkably elevated between 15 to 60 min, and then remained more or less invariant at 120 min in comparison of control, whereas phosphorylated Yap was declined at different time course (Figure 1E). Furthermore, after introduction of 8xGTIIC-luciferase plasmid which contained more Tead binding sites and was extensively used to determine Yap-Tead transcriptional activity 5,9, luciferase activity presented an obvious elevation, except an undistinguishable change in comparison of control at 120 min (Figure 1F).

### Yap function in stromal cell proliferation and differentiation

**Figure 2:**
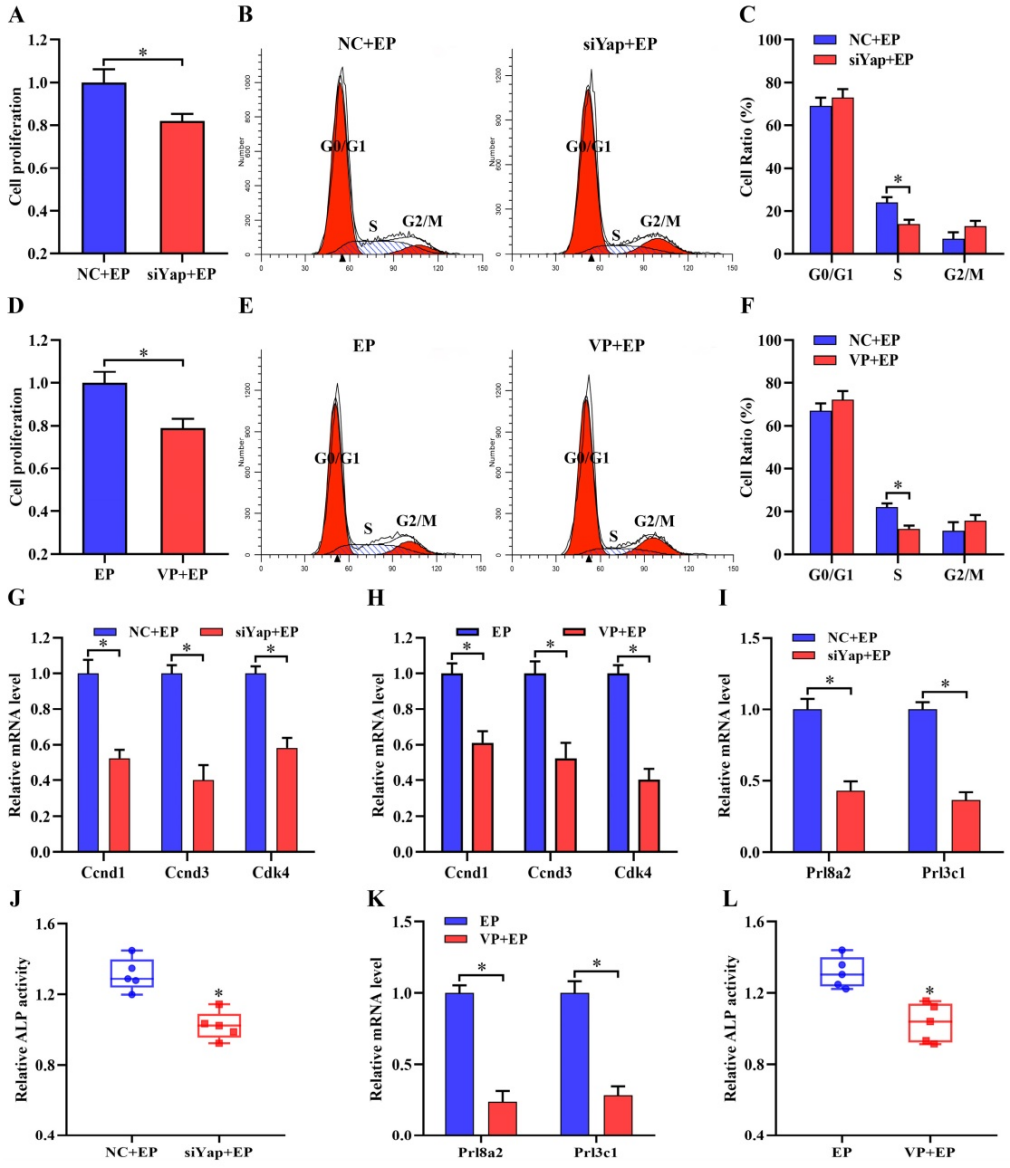
** Yap function in stromal cell proliferation and differentiation. (A)** Effect of Yap siRNA on stromal cell proliferation (N = 6 per group). NC, negative control; siYap, Yap siRNA. **(B and C)** Effect of Yap siRNA on cell cycle (N = 3 per group). **(D)** Effect of Yap inhibitor Verteporfin on stromal cell proliferation (N = 6 per group). VP, Verteporfin. **(E and F)** Effect of Yap inhibitor Verteporfin on cell cycle (N = 3 per group). **(G and H)** Regulation of Yap siRNA or Verteporfin on the expression of *Ccnd1*, *Ccnd3* and* Cdk4* (N = 5 per group). **(I and J)** Knockdown of Yap diminished the expression of *Prl8a2* and *Prl3c1* as well as ALP activity (N = 5 per group). **(K and L)** Verteporfin restrained the expression of *Prl8a2* and *Prl3c1* as well as ALP activity (N = 5 per group). Data are shown mean ± SEM. Asterisks denote significance (P < 0.05).

To determine the effects of Yap on stromal cell proliferation which was the first step of uterine decidualization 1, Yap siRNAs were delivered into stromal cells followed by the analysis of MTS and flow cytometry. The results demonstrated that after introduction of Yap siRNA 1, 2 and 3, which effectively restrained its mRNA and protein levels as well as Yap-Tead transcription activity along with obvious repressive action for Yap siRNA 3, cell proliferation was weakened concomitant with a low transition of cell cycle from G1 stage into S phase (Figure 2A-C, Figure S2A-C). Similarly, addition of Yap inhibitor Verteporfin, which disrupted Yap-Tead interaction with a reduction of its transcription activity, resulted in an obvious attenuation for stromal cell proliferation and impeded the progression of cell cycle from G1 through S phase (Figure 2D-F, Figure S2D). Further analysis evidenced that inactivation of Yap by specific siRNA and Verteporfin suppressed the expression of cyclin D1 (*Ccnd1*), *Ccnd3* and cyclin-dependent kinase 4 (*Cdk4*) which were principal regulators in G1/S phase transition 19 (Figure 2G and H), but did not alter mRNA levels of *Ccna1*, *Ccnb1*, *Ccnb2*, *Ccne1*, *Cdk1*, *Cdk2* and *Cdk6* (Data not shown).

To assess Yap importance in stromal cell differentiation, we tested its influence on mRNA levels of prolactin family 8, subfamily a, member 2 (Prl8a2) and prolactin family 3, subfamily c, member 1 (Prl3c1) as well as alkaline phosphatase (ALP) activity, well-established stromal differentiation markers 15. Knockdown of Yap lessened the expression of *Prl8a2* and *Prl3c1*, and restrained the activity of ALP (Figure 2I and J). Concurrently, replenishment of Yap inhibitor Verteporfin caused a distinct impediment for stromal differentiation (Figure 2K and L).

### Yap mediated the regulation of Bmp2 on stromal differentiation

**Figure 3:**
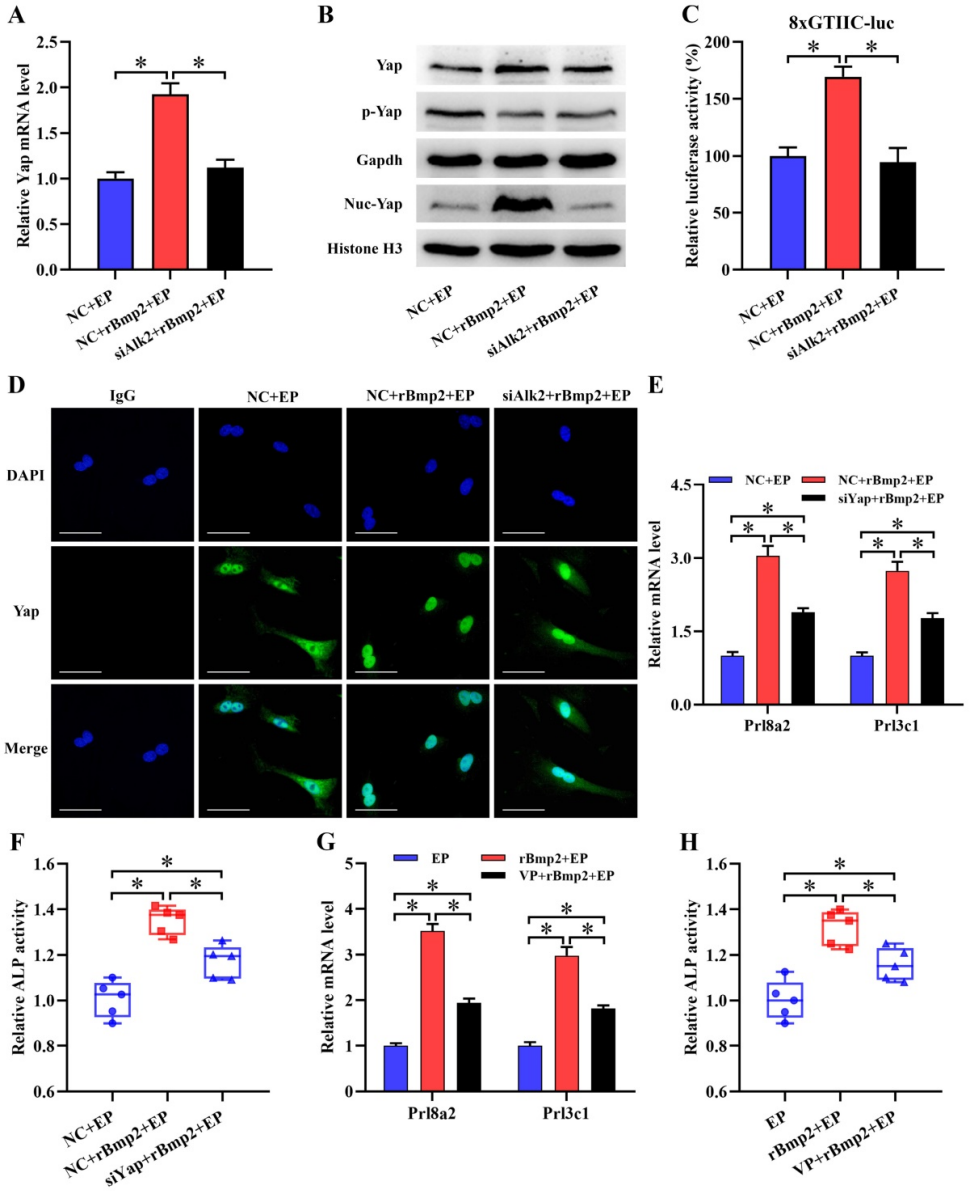
** Yap mediated the regulation of Bmp2 on stromal differentiation. (A and B)** Real-time PCR and western blot analyses of Yap expression after treatment with rBmp2 in the existence or not of Alk2 siRNA (N = 3 per group). siAlk2, Alk2 siRNA. **(C)** Bmp2 enhanced the Yap-Tead transcription activity via Alk2 (N = 6 per group). **(D)** Immunofluorescence analysis of Yap expression after treatment with rBmp2 in the absence or presence of Alk2 siRNA (N = 3 per group). Scale bar, 60 µm. **(E and F)** Knockdown of Yap blocked the induction of Bmp2 on *Prl8a2* and *Prl3c1* expression as well as ALP activity (N = 5 per group). **(G and H)** Verteporfin hampered the induction of Bmp2 on *Prl8a2* and *Prl3c1* expression as well as ALP activity (N = 5 per group).

It is well known that Bmp2 is an important regulator of uterine decidualization 3,4. Addition of rBmp2 markedly increased mRNA level of Yap along with slight alteration for its total and phosphorylated protein during *in vitro* decidualization (Figure 3A and B). Interestingly, Bmp2 drastically enhanced the expression of nuclear Yap protein and advanced Yap-Tead transcription activity (Figure 3B and C). Further analysis of Yap protein by immunofluorescence presented the shift from cytoplasm to nucleus after treatment with rBmp2 (Figure 3D). But knockdown of Bmp type I receptor Alk2 could abrogate the up-regulation of *Yap* mRNA and counteracted nuclear translocation of Yap protein concomitant with the deceleration of Yap-Tead transcription activity in rBmp2-treated stromal cells under *in vitro* decidualization (Figure 3A-D). We next wondered whether Yap might involve the regulation of Bmp2 in stromal differentiation. Administration of rBmp2 to decidualizing stromal cells treated with Yap siRNA or Verteporfin led to the abatement in the expression of differentiation markers such as *Prl8a2* and *Prl3c1* as well as ALP activity (Figure 3E-H).

### Rrm2 is direct target of Yap function in stromal differentiation

**Figure 4:**
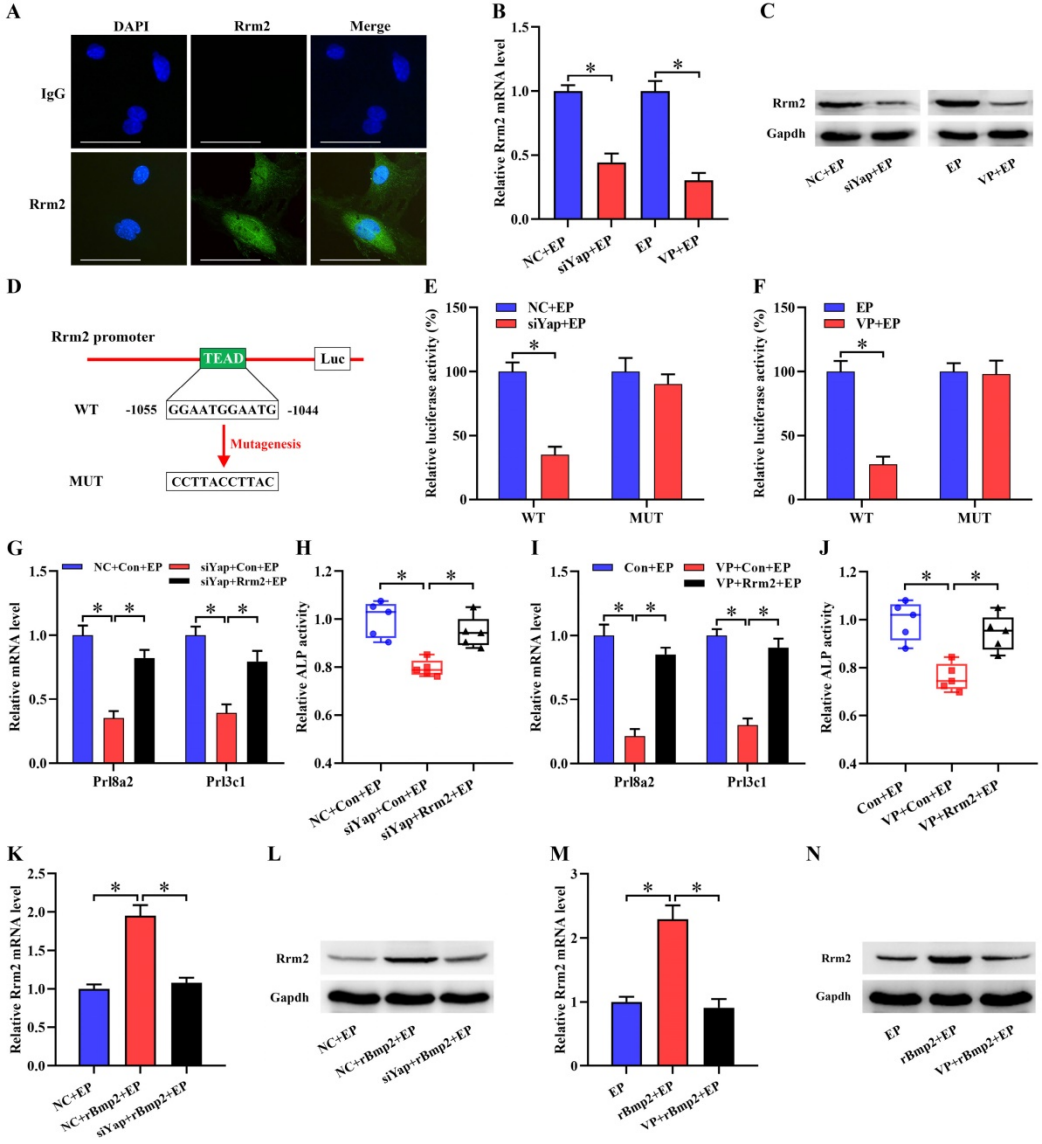
** Rrm2 was direct target of Yap function in stromal differentiation and its expression was regulated by Bmp2 dependent on Yap. (A)** Immunofluorescence analysis of Rrm2 expression in decidual cells (N = 3 per group). Scale bar, 60 µm. **(B and C)** Rrm2 mRNA and protein expression after treatment with Yap siRNA or Verteporfin (N = 3 per group). **(D)** Schematic diagram exhibited Yap/Tead binding sites and mutation in Rrm2 promoter region. **(E)** Relative luciferase activity after pGL6-Rrm2 or pGL6-Rrm2-mutant plasmid was co-transfected with Yap siRNA (N = 6 per group). **(F)** Relative luciferase activity after introduction of pGL6-Rrm2 or pGL6-Rrm2-mutant plasmid along with an addition of Yap inhibitor Verteporfin (N = 6 per group). **(G-J)** Overexpression of Rrm2 rescued the defect of *Prl8a2* and *Prl3c1* expression as well as ALP activity elicited by Yap inactivation (N = 5 per group). **(K-N)** Blockage of Yap abrogated the regulation of Bmp2 on Rrm2 mRNA and protein expression (N = 3 per group).

It has been previously reported that Rrm2 exerts an essential role in uterine decidualization 20. Rrm2 was abundantly visualized in decidual cells where its expression exhibited an overt decline after treatment with Yap siRNA or Verteporfin (Figure 4A-C). Further analysis displayed the presence of Tead binding site in Rrm2 promoter region (Figure 4D). After Rrm2 promoter-luciferase reporter plasmid was co-transfected with Yap siRNA, luciferase activity exhibited a remarkable decline (Figure 4E). Accordingly, blockage of Yap by Verteporfin restricted Rrm2 promoter-luciferase activity (Figure 4F). But mutation of Tead binding site gave rise to an incapability of Yap in repressing luciferase activity (Figure 4E and F). We next dissected whether Rrm2 might mediate the effects of Yap on stromal differentiation. Sustained activation of Rrm2, which brought about an apparent boost for corresponding mRNA and protein, rescued the defect of* Prl8a2* and *Prl3c1* expression as well as ALP activity elicited by the blockage of Yap (Figure 4G-J, Figure S3A and B).

### Yap mediated Bmp2 regulation of Rrm2

Under *in vitro* decidualization, complementarity of exogenous rBmp2 facilitated the up-regulation of Rrm2 mRNA and protein, but knockdown of Alk2 neutralized this up-regulation (Figure S3C and D). When Rrm2 inhibitor Triapine was appended, induction of Bmp2 on *Prl8a2* and *Prl3c1* expression as well as ALP activity was distinctly impeded (Figure S3E and F). Together these data evidenced Rrm2 as downstream target of Bmp2 in regulating stromal differentiation. We next explored the involvement of Yap in the regulation of Bmp2 on Rrm2. After delivery of Yap siRNA or Verteporfin, Bmp2 presented an inability in inducing the expression of Rrm2 (Figure 4K-N).

### Inactivation of Yap causes oxidative DNA damage via Rrm2-GSH-ROS pathway

**Figure 5:**
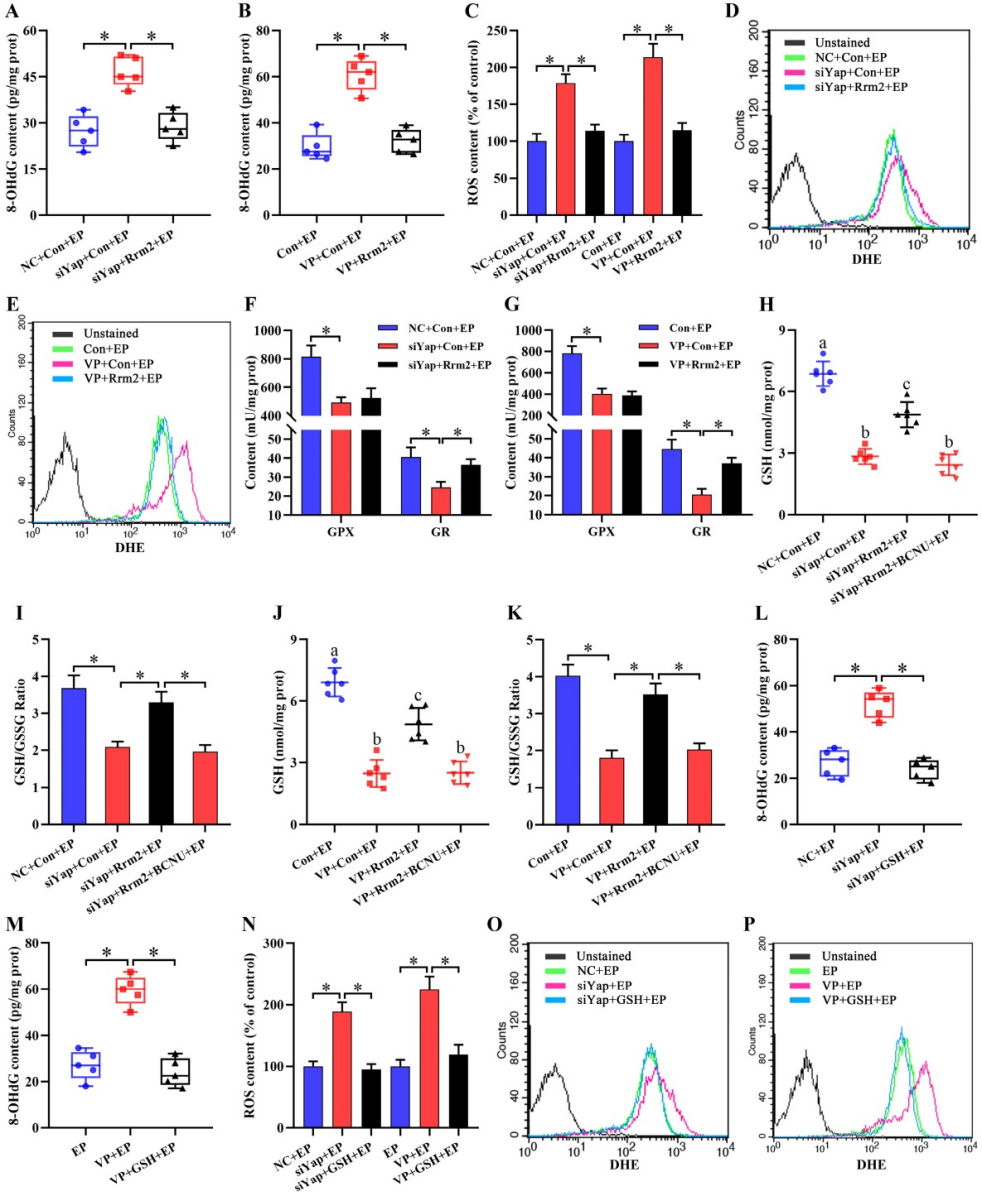
** Inactivation of Yap caused oxidative DNA damage via Rrm2/GSH/ROS pathway. (A and B)** Overexpression of Rrm2 impeded the enhancement of 8-OHdG content caused by Yap inactivation (N = 5 per group). **(C-E)** Overexpression of Rrm2 attenuated accumulation of intracellular ROS and O_2_^-^ by Yap inactivation (N = 3 per group). **(F)** Determination of GPX and GR antioxidant enzyme activities after co-transfection with Yap siRNA and Rrm2 overexpression plasmid (N = 4 per group). **(G)** Determination of GPX and GR antioxidant enzyme activities after transfection with Rrm2 overexpression plasmid along with an addition of Yap inhibitor Verteporfin (N = 4 per group). **(H-K)** Overexpression of Rrm2 resisted this alleviation of GSH content and GSH/GSSG ratio by Yap inactivation, but this resistance was abrogated by GR inhibitor BCNU (N = 6 per group). **(L and M)** GSH counteracted the elevation of 8-OHdG content generated by Yap inactivation (N = 5 per group). **(N-P)** GSH antagonized this accumulation of intracellular ROS and O_2_^-^ caused by Yap inactivation (N = 3 per group).

Previous evidence suggested the importance of Rrm2 in DNA damage repair 20. Because of the regulation of Yap on Rrm2, we hypothesized the potential involvement of Yap in DNA damage. As expected, impediment of Yap caused the elevation of 8-OHdG content which was widely used as a sensitive biomarker for oxidative DNA damage 21, whereas sustained activation of Rrm2 improved this anomalous 8-OHdG level (Figure 5A and B). It is well-known that ROS may induce DNA damage in exceeding the capacity of cellular antioxidant defenses 22,23. Inactivation of Yap gave rise to an obvious accumulation of intracellular ROS and O_2_^-^ concomitant with the defective activities of antioxidant enzyme GPX and GR, while overexpression of Rrm2 antagonized this accumulation, and rescued the activity of GR, but not GPX (Figure 5C-G). Consistently, blockage of Yap alleviated GSH content and GSH/GSSG ratio, while constitutive activation of Rrm2 resisted this alleviation (Figure 5H-K). Furthermore, supplementation of GR inhibitor BCNU neutralized the rescue of Rrm2 activation on GSH content as well as GSH/GSSG ratio in Yap-inactivated stromal cells under *in vitro* decidualization (Figure 5H-K).

**Figure 6:**
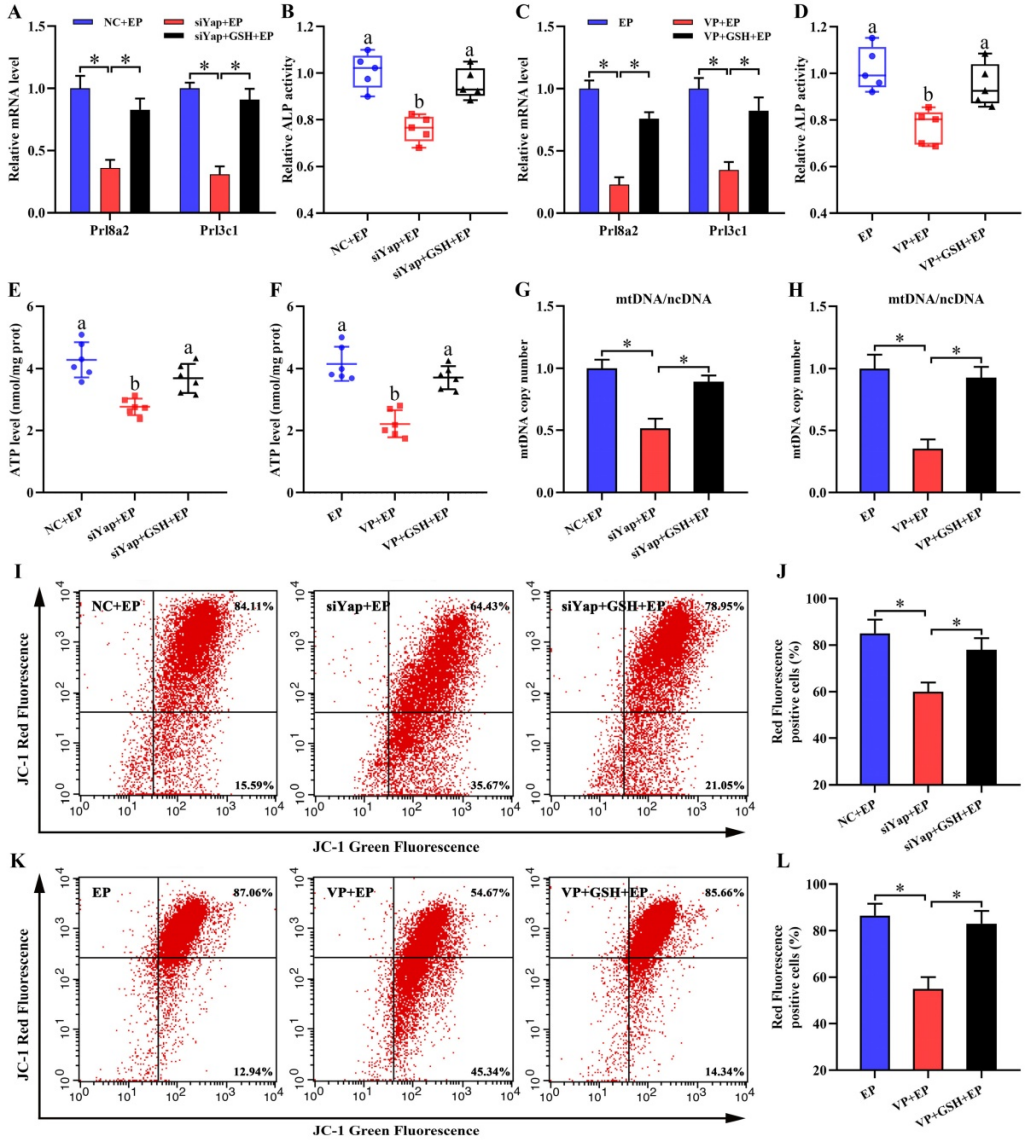
** Yap prevented mitochondrial dysfunction via GSH. (A-D)** GSH rescued the defect of *Prl8a2* and *Prl3c1* expression (N = 3 per group) as well as ALP activity (N = 5 per group) caused by Yap inactivation. **(E-H)** ATP level (N = 6 per group) and mtDNA copy number (N = 3 per group) were determined after treatment with Yap siRNA or Verteporfin along with an addition of GSH. **(I-L)** Flow cytometry analysis of MMP after treatment with Yap siRNA or Verteporfin and then addition of GSH (N = 3 per group).

To dissect whether GSH was sufficient to ameliorate oxidative DNA damage and aberrant stromal differentiation caused by blockage of Yap, uterine stromal cells were replenished with exogenous GSH in the existence of Yap siRNA or Verteporfin under the context of estrogen and progesterone. The results found that GSH might counteract the elevation of 8-OHdG content generated by Yap inactivation together with the attenuation for intracellular ROS and O_2_^-^ levels (Figure 5L-P). Concurrently, addition of GSH reversed the down-regulation of *Prl8a2* and *Prl3c1* expression by Yap blockage and rescued the deficit of ALP activity (Figure 6A-D).

### Inactivation of Yap causes mitochondrial dysfunction and stromal cell apoptosis

**Figure 7:**
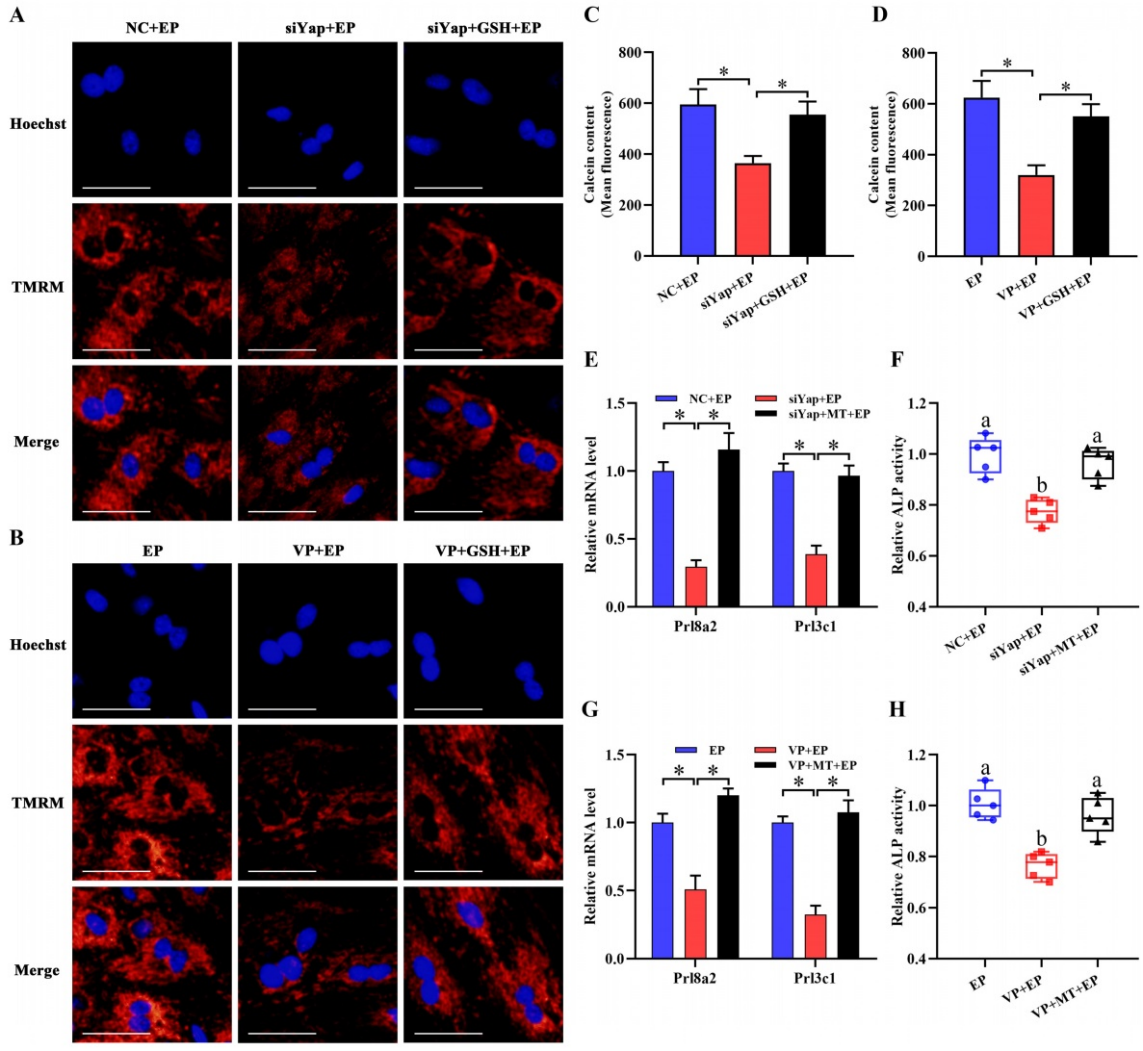
** Improvement of mitochondrial function rescued the defect of stromal differentiation caused by Yap inactivation. (A and B)** MMP visualization after treatment with Yap siRNA or Verteporfin and then addition of GSH (N = 3 per group). Scale bar, 60 µm.** (C and D)** Flow cytometry analysis of mPTP after treatment with Yap siRNA or Verteporfin along with an addition of GSH (N = 3 per group). **(E-H)** Mito-TEMPO rescued the fault of *Prl8a2* and *Prl3c1* expression (N = 3 per group) as well as ALP activity (N = 5 per group) elicited by Yap inactivation. MT, Mito-TEMPO.

Mitochondria are necessary to maintain cellular function by orchestrating energy production 24,25. Inactivation of Yap lessened intracellular ATP content and brought about the aberration of mtDNA copy number, but this abnormality was rescued by GSH (Figure 6E-H). It has been established that MMP definitely reflects mitochondrial function 26. After introduction of Yap siRNA or addition of Verteporfin, MMP denoted an obvious decline as evinced by the reduction for red/green fluorescence ratio, but this decline was obstructed by GSH (Figure 6I-L). To further illustrate Yap role in the maintenance of MMP, we used another indicator TMRM and found that blockage of Yap resulted in an attenuation of fluorescence signal, whereas adjunction of GSH recovered this fluorescence intensity (Figure 7A and B). Simultaneously, repression of Yap gave rise to the opening of mPTP, but exogenous GSH prevented this opening (Figure 7C and D). Further analysis indicated that administration of mitochondria-targeted antioxidant Mito-TEMPO rescued the fault of stromal differentiation elicited by the blockage of Yap as indicated by the amelioration for differentiation markers *Prl8a2*, *Prl3c1* and ALP (Figure 7E-H).

**Figure 8:**
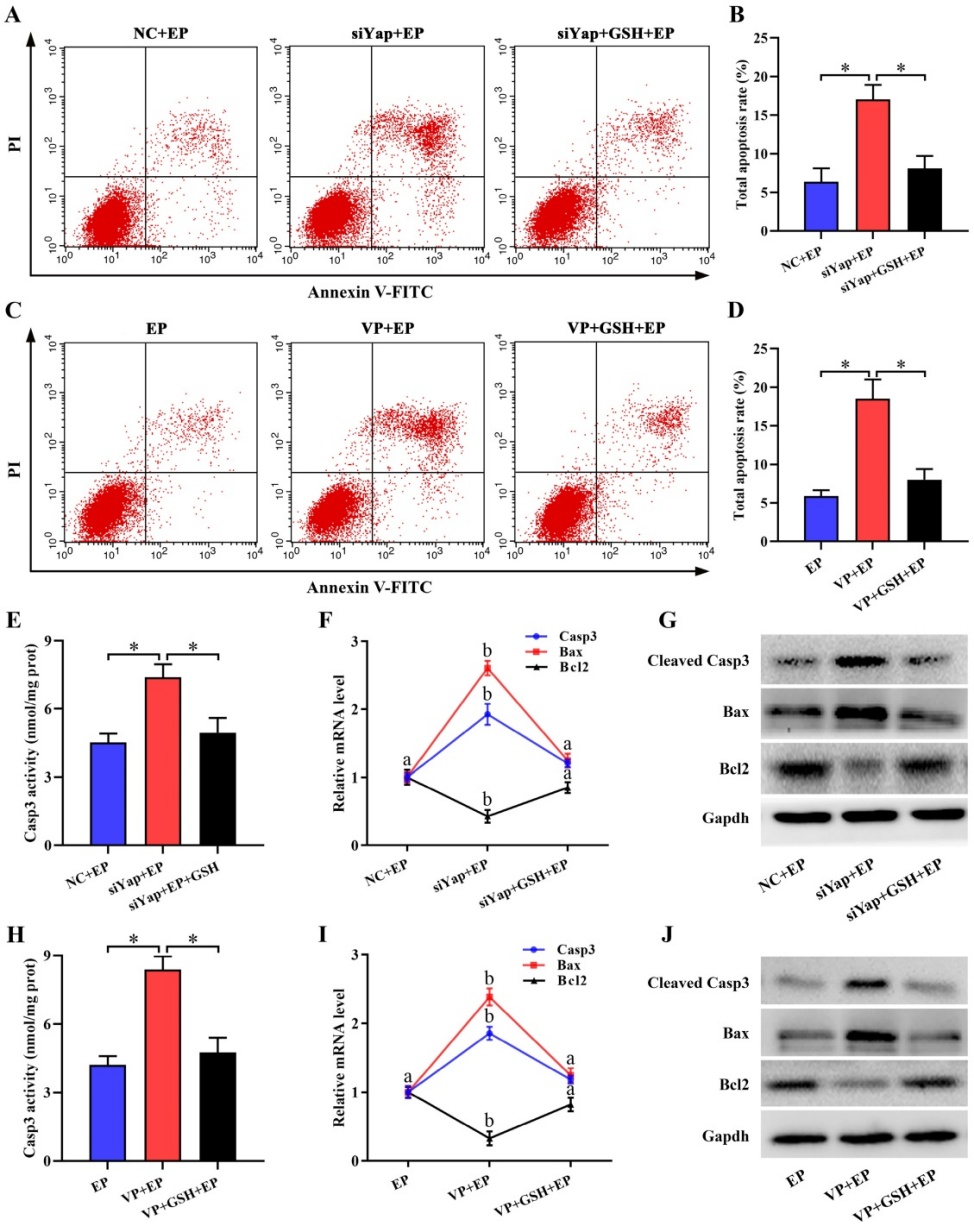
** Inactivation of Yap triggered stromal cell apoptosis. (A-D)** Flow cytometry analysis of cell apoptosis after treatment with Yap siRNA or Verteporfin in the existence or not of GSH (N = 3 per group). **(E-G)** Determination of Casp3, Bax and Bcl2 expression (N = 3 per group) as well as Casp3 activity (N = 6 per group) after introduction of Yap siRNA and then addition of GSH. **(H-J)** Determination of Casp3, Bax and Bcl2 expression (N = 3 per group) as well as Casp3 activity (N = 6 per group) after treatment with Yap inhibitor Verteporfin along with an addition of GSH.

Mitochondria have been proved as a crucial trigger of cell apoptosis 27,28. Suppression of Yap enhanced the apoptosis of stromal cells under *in vitro* decidualization, but this enhancement was disrupted by GSH (Figure 8A-D). To reveal the underlying cause of pro-apoptosis resulted from Yap inactivation, we monitored its regulation on Casp3, Bcl2 and Bax. The results demonstrated that blockage of Yap strengthened Casp3 activity and up-regulated mRNA levels for *Casp3* and *Bax*, and promoted the expression of cleaved Casp3 and Bax protein followed by a remarkable diminishment of anti-apoptotic Bcl2 expression, while replenishment of GSH recovered aberrant expression or activity for Casp3, Bax and Bcl2 in Yap-inactivated stromal cells (Figure 8E-J).

## Discussion

Yap is crucial for the development of ovarian follicle and early embryo 9-12, but there is limited data regarding its role in uterine decidualization. The present study showed the abundant Yap in decidual cells, suggesting a potential function for Yap in decidualization which was characterized by the proliferation and differentiation of stromal cells 1. Inhibition of Yap obviously restricted the proliferation of stromal cells. It is well-known that proliferation relies on cell cycle which is processed through G0/G1, S, G2 and M phases and orchestrated by cyclins in association with Cdks 19,29. Blockage of Yap resulted in a slow progression from G1 into S phase and down-regulated the expression of *Ccnd1*, *Ccnd3* and *Cdk4*, indicating an importance of Yap in governing G1/S phase transition, which was further highlighted by these evidences that sustained activation of Yap accelerated the transition of podocytes and cardiomyocytes to enter into S phase, whereas depletion of Yap in KYSE170 cells and malignant mesothelioma cells caused cell cycle arrest at G1 phase 30-33. Followed by the initiation of proliferation, uterine stromal cells differentiated into decidual cells 1. Inactivation of Yap hampered stromal differentiation as evidenced by the reduction for differentiation markers *Prl8a2* and *Prl3c1* expression as well as ALP activity. Together these observations suggest that Yap is required for uterine decidualization.

Bmp2 is of great importance to decidualization program, because its deficiency led to an abnormality of decidual reaction 3,4. Under *in vitro* decidualization, Bmp2 enhanced the levels of Yap mRNA and nuclear protein, but this enhancement was annihilated by siRNA against Bmp type 1 receptor Alk2 whose ablation resulted in compromised decidualization 34. Suppression of Yap attenuated the irritation of Bmp2 on stromal differentiation, implying that Yap might be a downstream target of Bmp2, which was reinforced by these data that deficiency of Yap hindered the induction of astrocytic differentiation by Bmp2 and abrogated the regulation of Bmp2 on the proliferation of embryonic neural stem cells 35,36. Further analysis demonstrated that Bmp2 promoted Yap nuclear translocation where unphosphorylated Yap might drive the expression of downstream genes in interaction with DNA-binding Tead transcription factors 6,7. In decidualizing stromal cells, Yap adjusted the expression of Rrm2 which was a rate-limiting enzyme for the production of dNTP required for DNA synthesis, and its blockage impaired decidualization 18. Concurrently, Rrm2 promoter region presented the existence of Yap/Tead binding site whose mutation abrogated their integration. Overexpression of Rrm2 ameliorated the defect of stromal differentiation caused by Yap inactivation. Together these data reveal that Rrm2 is a direct target of Yap in uterine decidualization. It has been previously reported that after binding to transmembrane receptor, Bmp2 may activate Smad1/5/8 to modulate various gene expression in the process of osteogenic differentiation via cooperative interaction with Yap 37. In decidualizing stromal cells, inactivation of Yap antagonized the induction of Bmp2 on Rrm2 expression, indicating YAP as a crosstalk junction between Bmp2 and Rrm2. However, it is still to be determined regarding the interplay between Smad1/5/8 and Yap in decidualization.

ROS were the byproducts of cellular aerobic metabolism and its accumulation impaired uterine decidualization after exceeding the capacity of cellular antioxidant defenses 38. Under *in vitro* decidualization, inactivation of Yap gave rise to an obvious accumulation of intracellular ROS. GSH was regarded as the most important antioxidant to scavenge ROS by oxidation to GSSG dependent on GPX whose repression by mercaptosuccinic acid resulted in a reduction of pregnancy rate along with the defective stromal differentiation 39,40. GSSG was subsequently converted back to GSH by GR to reuse in cells 40. After exposure to siRNA against Yap or Verteporfin, faultinesses for GSH content as well as GPX and GR activities were noted in decidualizing stromal cells, but sustained activation of Rrm2 improved GSH level and rescued the defective activity of GR whose inhibition by BCNU neutralized the rescue of Rrm2 activation on GSH content. Replenishment of GSH ameliorated the defect of stromal differentiation caused by Yap blockage along with the reduction of intracellular ROS. Taken together, these observations indicate that cause by which Yap inactivation impairs uterine decidualization contributes to the overaccumulation of ROS dependent on defective Rrm2-GR-GSH pathway. Additionally, overproduction of ROS led to DNA damage 22,23. Impediment of Yap brought about the elevation of 8-OHdG content which was a sensitive biomarker for oxidative DNA damage, but this elevation was hampered by GSH, implying Yap as redox modulator against DNA injury.

Mitochondria are powerhouse organelles implicated in the control of energy production 24,25. Under *in vitro* decidualization, inactivation of Yap led to an obvious decline of ATP level, a powerful indicator of mitochondrial function 15. Owing to the lack of protective histones, mtDNA was vulnerable to ROS and its depletion resulted in the diminishment of MMP which generated by proton pumps and formed the transmembrane potential of hydrogen ions that were required for ATP synthesis 25,26,41. Blockage of Yap gave rise to the aberration of mtDNA copy number and MMP, but this abnormality was rescued by GSH, implying an importance of Yap in preventing mitochondrial dysfunction whose improvement by Mito-TEMPO rescued the fault of stromal differentiation elicited by Yap inactivation. Further analysis declared that mitochondrial damage brought about the long-lasting opening of mPTP which caused the release of proapoptotic factors into the cytosol to trigger cell apoptosis 28,42. Pro-apoptotic effector Bax induced mPTP opening of long duration, whereas anti-apoptotic Bcl2 maintained the integrity of mitochondria 28,43. Inactivation of Yap gave rise to an obvious enhancement for mPTP opening and stromal cell apoptosis through the induction of executioner Casp3 followed by a remarkable elevation of Bax expression and reduction for Bcl2, but this effectiveness was improved by GSH, indicating Yap as an important regulator in impeding stromal cell apoptosis during decidualization.

## Conclusions

Yap is necessary for uterine decidualization in respond to Bmp2. Inactivation of Yap results in an abundant accumulation of intracellular ROS via defective Rrm2/GR/GSH pathway, which further brings about DNA damage, mitochondrial dysfunction and cellular apoptosis.

## Supplementary Material

Supplementary figures and table.
