# Supplementary material for: Yap is essential for uterine decidualization through Rrm2/GSH/ROS pathway in response to Bmp2
Source: Int J Biol Sci. 2022 Mar 6;18(6):2261–76. doi: 10.7150/ijbs.67756 (PMC8990470; doi:10.7150/ijbs.67756)
Supplement: Supplementary file 1 — Supplementary figures and table. [file ijbsv18p2261s1.pdf]

Figure S1

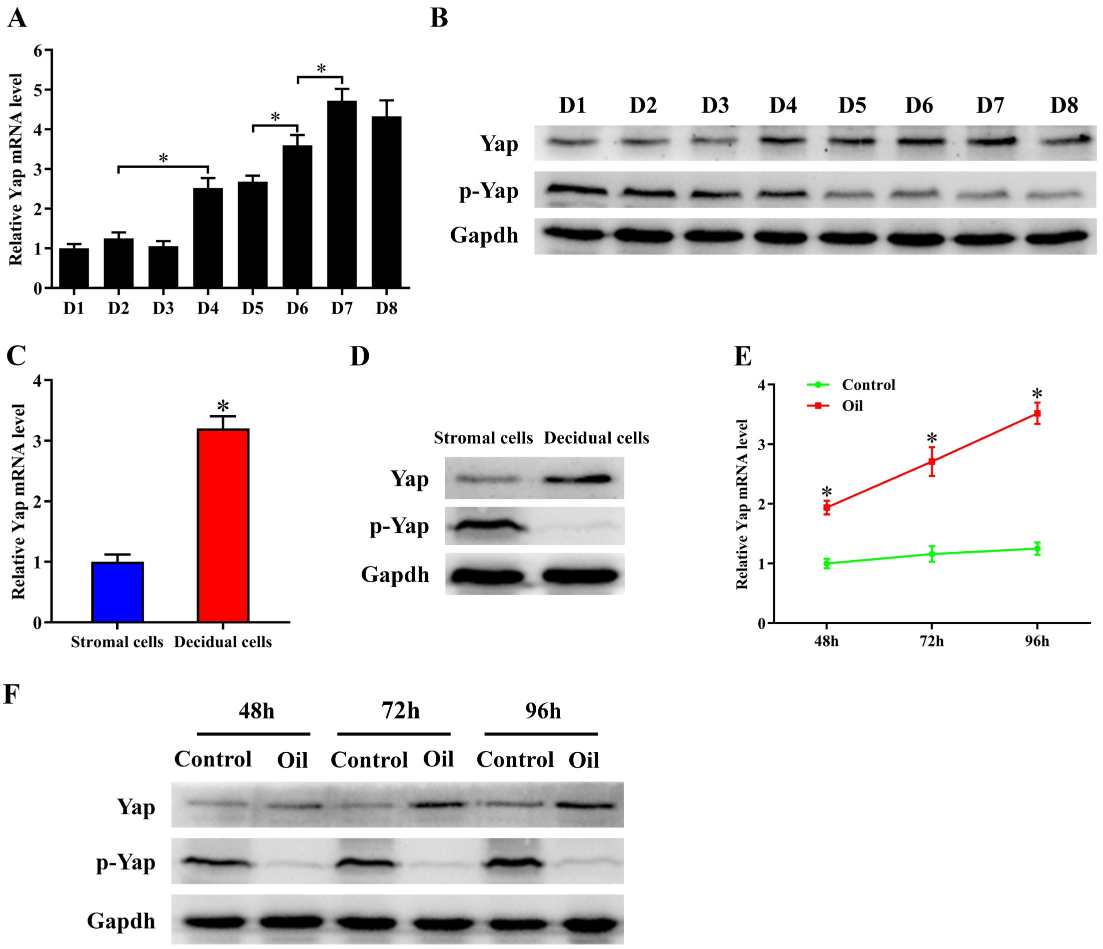

Figure S1. Yap expression during decidualization. (A) and (B) Real-time PCR and western blot analyses of Yap expression in mice uteri on days 1-8 of pregnancy (3 mice per group). (C) and (D) Yap mRNA and protein expression in decidual and stromal cells (3 mice per group). (E) and (F) Yap mRNA and protein expression under artificial decidualization (3 mice per group). Data are shown mean ± SEM, Asterisks denote significance ($P < 0.05$).

Figure S2

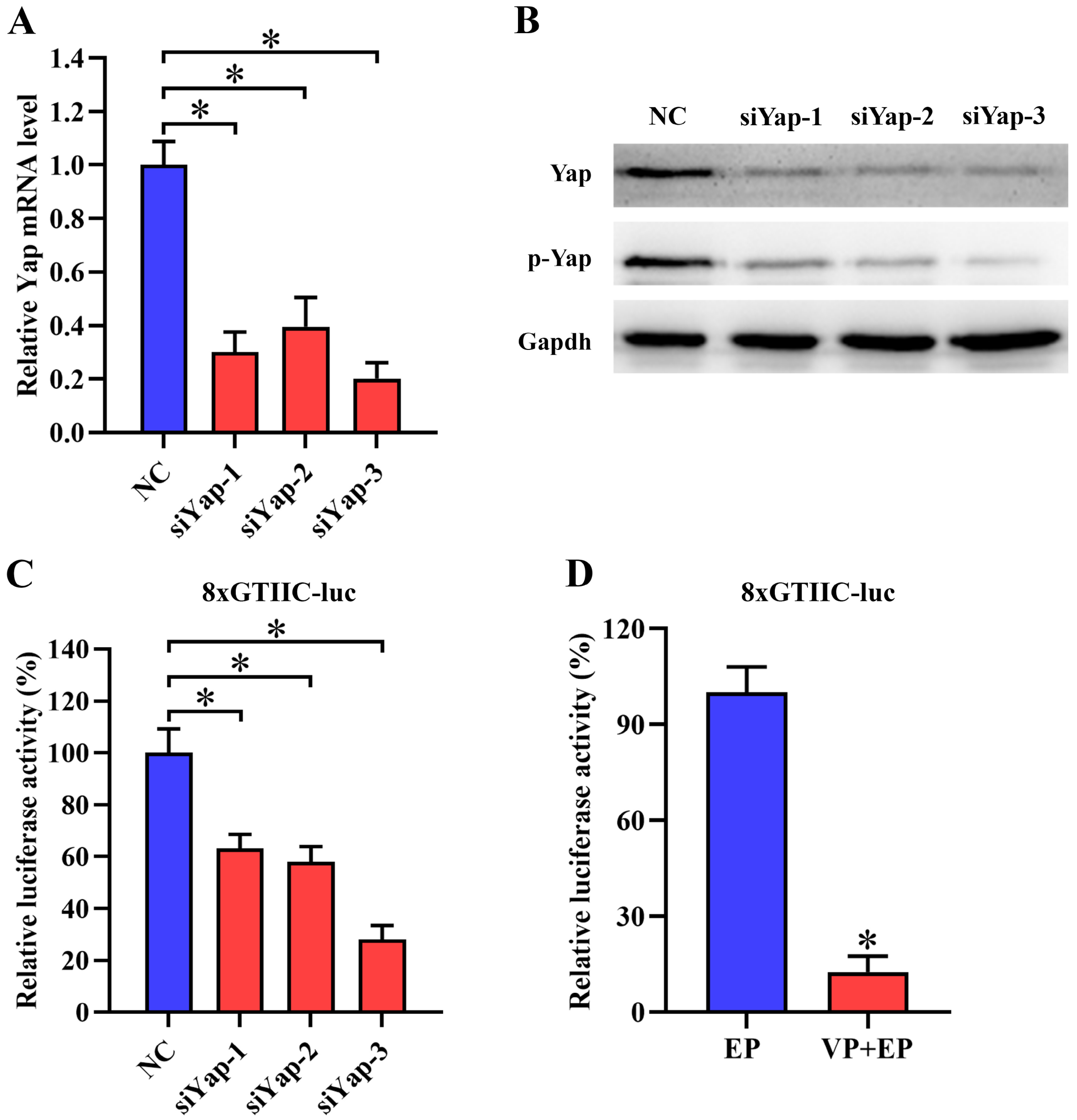

Figure S2. Yap mRNA and protein levels as well as Yap-Tead transcriptional activity in stromal cells after treatment with Yap siRNA or Verteporfin. (A-C) Yap mRNA and protein levels (N = 3 per group) as well as Yap-Tead transcriptional activity (N = 5 per group) in stromal cells after introduction of Yap siRNA. (D) Effect of Yap inhibitor Verteporfin on Yap-Tead transcriptional activity (N = 5 per group).

Figure S3

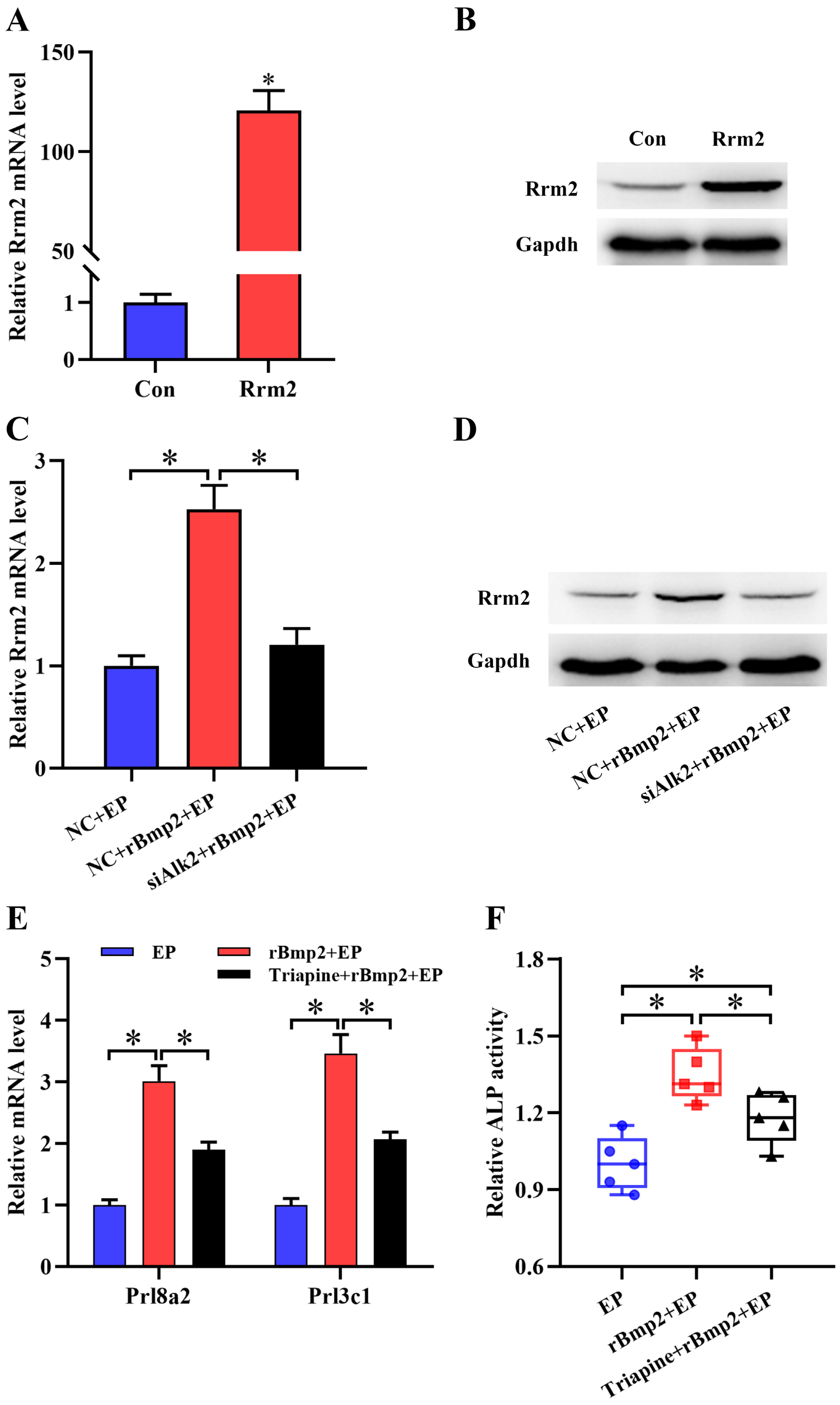

Figure S3. Rrm2 mediated the effect of Bmp2 on stromal differentiation. (A) and (B) Rrm2 mRNA and protein expression was evaluated after introduction of Rrm2 overexpression plasmid (N = 3 per group). (C) and (D) Rrm2 mRNA and protein expression was evaluated after exposure to rBmp2 with/without Alk2 siRNA (N = 3 per group). (E) and (F) Repression of Rrm2 by Triapine prevented the induction of Bmp2 on *Prl8a2* and *Prl3c1* expression (N = 3 per group) as

well as ALP activity (N = 5 per group).

Table S1. Primers used in this study

Gene	Sequence of Forward Primer	Sequence of Reverse Primer	Application
Yap	TTAACAGTGGCACCTATCAC	TCCAAGATTTCGGAACTCAG	In situ hybridization
Rrm2	AAGCTTATGCTCTCCGTCCGCACCCCG	CTCGAGTTAGAAGTCAGCATCCAAGGT	Overexpression
Bax	CCGGCGAATTGGAGATGAACT	CCAGCCCATGATGGTTCTGAT	Real-time PCR
Bcl2	TCAGAGCGAGAAGGTAGGGA	CTGTGGGGTAACAAGAAGGTC	Real-time PCR
Casp3	CTGGACTGTGGCATTGAGAC	GCAAAGGGACTGGATGAACC	Real-time PCR
Ccna1	GCCCGACGTGGATGAGTTT	AGGAGGAATTGGTTGGTGGTT	Real-time PCR
Ccnb1	CTGAGCCTGAGCCTGAACCT	AGCCCCATCATCTGCGTCT	Real-time PCR
Ccnb2	GCTAGCTCCCAAGGATCGTC	CTGCAGAGCTGAGGGTTCTC	Real-time PCR
Ccnd1	GGGATGTGAGGGAAGAGGTGA	GCAGCGAAAACAACGTGAAA	Real-time PCR
Ccnd3	CCTCCTACTTCCAGTGCGTG	GGCAGACGGTACCTAGAAGC	Real-time PCR
Ccne1	AATGGATGGTTCCGTTCGC	TGGGTCTGGATGTTGTGGG	Real-time PCR
Cdk1	CTGGGCACTCCTAACAACGAAG	TCCAAGCCGTTCTCGTCCAG	Real-time PCR
Cdk2	ACAGGGCAAGGTGAAAGAC	AGGAGGACGGCAATGAGG	Real-time PCR
Cdk4	GTGGCTGAAATTGGTGTCGG	TAACAAGGCCACCTCACGAA	Real-time PCR
Cdk6	TCCTGCTCCAGTCCAGCTAT	CCACGTCTGAACTTCCACGA	Real-time PCR
Prl8a2	AGCCAGAAATCACTGCCACT	TGATCCATGCACCCATAAAA	Real-time PCR
Prl3c1	GCCACACGATATGACCGGAA	GGTTTGGCACATCTTGGTGTT	Real-time PCR
mtDNA	CCTATCACCCTTGCCATCAT	GAGGCTGTTGCTTGTGTGAC	Real-time PCR
ncDNA	ATGGAAAGCCTGCCATCATG	TCCTTGTTGTTCAGCATCAC	Real-time PCR
Rrm2	AGTTCCTCACGGAGGCCTT	CTCTGATACTCGCCTACTCGC	Real-time PCR
YAP	CCGTTTCTCCTGGGACACTC	CTGCTGGCAGTGGTACATCA	Real-time PCR
